# Effects of Calcium Carbide Slag Incorporation on the Multiscale Performance of Sulfoaluminate Cement Mortars

**DOI:** 10.3390/ma19040746

**Published:** 2026-02-14

**Authors:** Jianqing Tang, Liaojun Zhang, Su Lu, Jiaxin Liu, Shuo Wang, Shasha Li, Jing Li, Zhongying Li

**Affiliations:** 1 College of Water Conservancy & Hydropower Engineering, Hohai University, Nanjing 210098, China; qing93@hhu.edu.cn (J.T.); lusu0821@hhu.edu.cn (S.L.); ljx1104@hhu.edu.cn (J.L.); qqwang@hhu.edu.cn (S.W.); 2 Department of Structural Geotechnical and Building Engineering, Politecnico di Torino, 10129 Torino, Italy; 3 Institute of Water Science and Technology, Hohai University, Nanjing 210098, China; 4 School of Qilu Transportation, Shandong University, Jinan 250002, China; lishasha0902@163.com; 5 Xinjiang College of Science and Technology, Korla 841000, China; 15247143014@163.com; 6 School of Civil and Resource Engineering, University of Science and Technology Beijing, Beijing 100083, China; lzy0119@xs.ustb.edu.cn

**Keywords:** calcium carbide slag, sulphoaluminate cement, electrochemical properties, microstructure, hydration behavior

## Abstract

This study investigated the effects of calcium carbide slag (CCS) (0–12 wt%) incorporation on the workability, electrochemical properties, durability, and microstructure evolution of sulfoaluminate cement (SAC) mortar. Results showed that increasing CCS content reduced mortar fluidity and shortened setting time, indicating that CCS accelerates early hydration. A 9% CCS content was determined to be the optimal dosage; at 28 days, compared to the control group, this dosage group exhibited a 6.53% increase in compressive strength, a 22.47% decrease in drying shrinkage, and a 0.279% decrease in mass loss. These performance improvements stemmed from CCS’s ability to inhibit pore connectivity and limit moisture migration. Electrochemical analysis further revealed that the 9% CCS dosage group had the highest charge transfer resistance and resistivity (30.00% higher than the control group), reflecting a denser matrix and greater ion transport resistance. Consequently, chloride ion permeability was significantly reduced, with electrical flux and diffusion coefficient decreasing by 39.98% and 28.89%, respectively. Microstructural observations confirmed that CCS promotes the formation and densification of hydration products, effectively improving the internal pore structure. While 9% CCS can serve as an effective functional supplementary material, its long-term durability and sustainability still face practical application challenges. Future research should focus on establishing predictive models for chloride ion permeation lifetime and conducting quantitative sustainability assessments of CCS-SAC composites, particularly evaluating material cost, energy consumption, and carbon dioxide emissions.

## 1. Introduction

With the continuous advancement of cementitious materials, the demand for low-carbon, durable, and high-performance binders in the construction industry have increased significantly. Among various cement types, sulfoaluminate cement (SAC) has attracted considerable attention due to its rapid setting, low alkalinity, fast early strength development, and excellent sulfate resistance, making it suitable for construction under diverse and demanding service environments. Compared with ordinary Portland cement (OPC), SAC exhibits substantially lower carbon dioxide (CO_2_) emissions owing to its lower limestone content and reduced calcination temperature, thereby offering clear environmental advantages [1,2,3]. The rapid hardening and early strength characteristics of SAC are primarily attributed to its high calcium aluminate content, which undergoes fast hydration to form ettringite (AFt) and aluminum hydroxide (AH_3_), resulting in rapid strength gain at early ages [4]. The relatively low clinker temperature and reduced CaCO_3_ consumption during SAC production further enhance its sustainability potential [5,6,7]. Despite these advantages, the rapid hydration kinetics of SAC also raise concerns regarding long-term hydration stability, volumetric stability, and durability, which remain critical challenges in practical engineering applications [8]. The fast-setting nature of SAC imposes restrictions on the incorporation of chemical and mineral admixtures, limiting its adaptability in complex construction scenarios [9]. The partial replacement of SAC with suitable industrial by-products has been widely considered an effective strategy to improve long-term performance while further reducing environmental impact [10,11].

Considering the relatively low pH environment of SAC systems, alkaline industrial residues are regarded as promising admixtures. Calcium carbide slag (CCS), an alkaline solid waste generated during acetylene gas production, is produced in large quantities and presents significant recycling potential [12]. CCS is characterized by high alkalinity, fine particle size, and latent pozzolanic reactivity, and it is mainly composed of Ca(OH)_2_ with minor amounts of CaCO_3_ and other reactive constituents [13]. When incorporated into SAC systems, CCS can release free Ca^2+^ ions and act as fine fillers, potentially promoting the formation of additional calcium (alumino) silicate hydrate (C-(A)-S-H) gels through secondary reactions, thereby refining the microstructure of hydrated products.

The high fineness and compositional variability of CCS often increase water demand, and its elevated Ca(OH)_2_ content may induce hydration-related expansion, which can result in microcracking and compromise long-term durability [14]. As a result, the introduction of CCS into SAC systems leads to complex chemical–physical interactions during hydration, and its effects on durability-related properties and microstructural evolution cannot be straightforwardly predicted. Systematic investigations are therefore required to clarify the coupled influence of CCS on hydration behavior, pore structure development, and long-term performance of SAC-based materials.

Previous studies have demonstrated that mineral admixtures such as fly ash (FA) [15], ground granulated blast furnace slag (GGBS), vermiculite [16,17], silica fume (SF), and limestone powder (LP) [18] can improve the workability, long-term strength, dimensional stability, and durability of SAC-based materials through filler effects, pozzolanic reactions, and modified AFt formation pathways [19]. In contrast, research on CCS-modified SAC systems remains relatively limited. Existing studies often focus on early hydration characteristics, setting behavior, or isolated strength properties, while comprehensive evaluations incorporating durability indicators and microstructure–property relationships are still scarce. In particular, the coupled effects of CCS on workability, shrinkage behavior, mechanical performance, electrochemical characteristics, chloride transport resistance, carbonation resistance, and microstructural evolution of SAC have not yet been fully elucidated.

The present study aims to systematically investigate the effects of CCS incorporation (0–12 wt%) on the fresh properties, mechanical behavior, electrochemical characteristics, durability performance, and microstructural evolution of SAC mortar. The evaluated parameters include workability (fluidity and setting time), flexural and compressive strengths at multiple curing ages, drying shrinkage and mass loss, electrical resistivity and electrochemical impedance spectroscopy (EIS), chloride ion penetration resistance and diffusion behavior, carbonation depth, and microstructural features observed by scanning electron microscopy (SEM). By establishing correlations between CCS content, hydration characteristics, microstructural development, and macroscopic performance, this study seeks to identify the compatibility and optimal dosage range of CCS in SAC systems, thereby providing scientific guidance for the sustainable utilization of industrial residues and the design of SAC-based materials with balanced early performance and long-term durability.

## 2. Materials and Methods

### 2.1. Materials

The constituent materials comprised SAC (Grade 42.5), CCS, ISO standard sand, and laboratory tap water; the SAC was supplied by Dengdian Group Cement Co., Ltd. (Dengfeng, China), with a surface area per unit mass of no less than 350 m^2^/kg; the CCS was sourced from Gongyi Longze Water Cleansing Materials Co., Ltd., Gongyi, China. X-ray fluorescence (XRF) analysis was conducted to determine the oxide compositions of SAC and CCS, with the results shown in Table 1, and their key performance indicators are presented in Table 2, which comply with the requirements of the industry standard JC/T 2282-2014 [20]. Laboratory tap water was employed in this study, while the sand was ISO standard sand provided by Xiamen ISO Standard Sand Co., Ltd., Xiamen, China. conforming to the national standard GB/T17671-2021 [21]. In addition, 0.3 mol/L NaOH solution and 3.0% NaCl solution were employed to test the diffusion coefficient and chloride ion electric flux of the specimens. All chemical reagents were acquired from Chengdu Kelong Chemical Co., Ltd., Chengdu, China. with a purity of over 99%.

**Figure 1:**
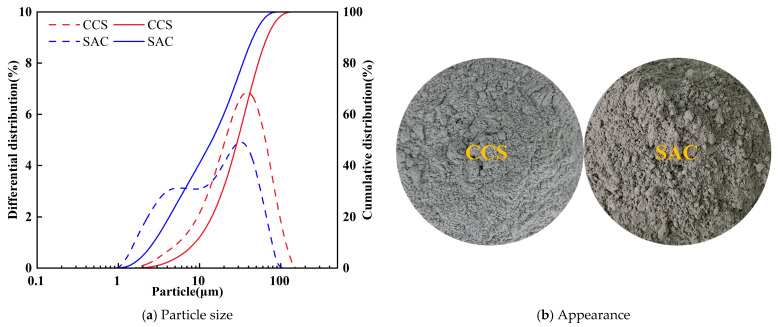
Appearance and particle size of CCS and SAC.

**Figure 2:**
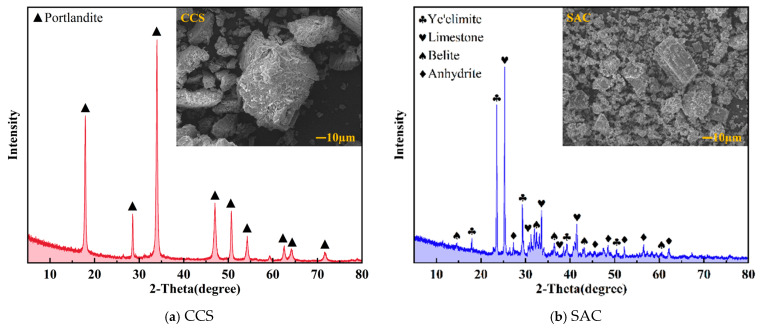
SEM observations and XRD patterns of CCS and SAC.

The size distribution of particles, appearance, mineral constituents, and microstructural characteristics of SAC and CCS used in this study are displayed in Figure 1 and Figure 2. Figure 1a compares the particle size distributions of SAC and CCS, revealing pronounced differences between the two materials: SAC has a relatively fine and wide particle size distribution with a high proportion of small-sized particles, whereas CCS features a relatively coarse particle size distribution dominated by medium- and large-sized particles. The cumulative distribution curves indicate that the grinding degree of CCS particles is generally lower than that of SAC, implying a smaller specific surface area for CCS. Figure 1b displays the appearance of the original powders: CCS has a lighter color and loose texture, while SAC appears denser and darker. X-ray diffraction (XRD) was performed, and the corresponding patterns are presented in Figure 2; there is a difference in composition between the two raw materials, SAC and CCS. The strong diffraction peaks in the XRD of CCS mainly correspond to Ca(OH)_2_. Other minor diffraction peaks of calcium-containing compounds can also be observed through XRD, indicating that Ca(OH)_2_ is the main crystalline phase of CCS. Additional microstructural features of the raw materials can be identified from the SEM images shown in Figure 2. CCS particles display a non-uniform shape with rough surfaces and porous structures, while SAC particles present a relatively angular shape and denser surfaces.

### 2.2. Specimen Design and Preparation

Based on the Chinese national standards GB 55008-2021, DL/T 5786-2019 and DL/T 5330-2015 [22,23,24], combined with relevant engineering practices, extensive preliminary tests and related prior studies, we designed an optimized mix proportion of SAC and CCS [13,14,25]. The CCS content was expressed as a mass fraction, and the mix ratios used in the specimen preparation are listed in Table 3. The CCS dosages selected in this study were as follows: 0% (denoted as CCS0, serving as the control group for comparison), 3% (CCS3), 6% (CCS6), 9% (CCS9) and 12% (CCS12). The mix proportions of all specimens were designed with a W/B ratio of 0.50 and a B/S ratio of 1:3.

To prepare fresh cement mortar, all solid materials were proportioned as designed and dry-mixed to achieve a homogeneous mixture. Subsequently, tap water was added and mixed to yield fresh cement mortar. The mixing procedure was as follows: Mixing was carried out by first blending the dry materials at low speed for 1 min, after which water was introduced and the mixture was mixed at low speed for 30 s and then at high speed for a further 30 s [26]. Upon completion of mixing, the workability test was conducted on the fresh cement mortar immediately. The fresh cement mortar was cast into moulds and subjected to vibration on a mortar vibration table for 40 s, after which the molds were removed and the surface of the specimens was screeded flat with a trowel promptly. The specimens were demoulded at 1 h ± 5 min starting from the moment water was added during mixing, and after casting, curing was carried out in a standard chamber at (20 ± 2) °C and >95% relative humidity until the target ages were reached.

### 2.3. Test and Characterization Methods

#### 2.3.1. Workability Test Method

Fluidity testing was conducted following the procedures specified in GB/T 2419-2005 (Test Method for Fluidity of Cement Mortars) [26,27] for the purpose of evaluating the fluidity and workability of the mortar. A truncated cone mold (with an upper diameter of 70 mm, a lower diameter of 100 mm, and a height of 60 mm) was adopted for the test. After lifting the mold, the mortar spread freely on the flow table. The final spread diameter (mm) was determined by averaging the values measured in two orthogonal directions.

The setting time test was performed following GB/T 1346-2024 (Test Methods for Water Requirement of Normal Consistency) [26,28], Setting Time and Soundness of the Cement. Initial and final setting times were measured with a Vicat apparatus after the freshly prepared mortar was poured into a standard mold. The penetration depth was determined at predetermined time intervals. The initial setting time was documented when the distance between the needle tip and the base plate attained 4 ± 1 mm, while the final setting time was determined when the needle tip could not penetrate into the mortar.

All workability measurements were conducted on at least three replicate specimens for each mixture. The reported values represent the average results, and the experimental uncertainty is expressed in terms of standard deviation.

#### 2.3.2. Mechanical Properties Test Method

The strength test of the mortar was conducted in accordance with the national standard GB/T 17671-2021 (Test Method for Strength of Cement Mortars (ISO Method)) [21,26], using 40 mm × 40 mm × 160 mm prisms as test samples. The mechanical properties were measured with a computer-controlled electro-hydraulic servo testing machine (HCT506C). The flexural fixture and compressive fixture were placed between the upper and lower pressure plates of the testing machine and aligned along the same axis as the machine. The loading rates for flexural and compressive tests were 50 ± 10 N/s and 2400 ± 200 N/s, and tests were conducted at curing ages of 6 h, 1, 3, 7, 14, and 28 d. At each curing age, the average of the flexural strength results of a set of three prisms is taken as the test result. If any of the three strength values exceeds ±10% of the average, it should be discarded and the average value should be taken as the flexural strength test result; if two of the three strength values exceed ±10% of the average, the remaining one is taken as the flexural strength result. Individual flexural strength results are accurate to 0.1 MPa, and the arithmetic mean is accurate to 0.1 MPa [21]. The flexural strength test employed a 3-point bending arrangement with a span length of 100 mm. A universal testing apparatus equipped with a three-point loading fixture was employed to apply the load at a controlled rate of (50 ± 10) N/s until the specimen fractured. The flexural strength $f_{f}$ was determined by means of Equation (1) provided below:

(1) $$f_{f}\;=\;\frac{3F_{f}L}{2bh^{2}}$$

where $F_{f}$ is the maximal imposed load (N), L is the span length (100 mm), and h and b are the height and width of the samples (both 40 mm).

The test result is the average of six compressive strength measurements obtained from three prisms. If any of the six measurements exceeds ±10% of the average, that result is discarded, and the average of the remaining five is taken as the final result. If any of the five measurements also exceeds ±10% of their average, the entire set of results is invalid. If two or more of the six measurements simultaneously exceed ±10% of the average, the entire set of results is invalid. Individual compressive strength results are accurate to 0.1 MPa, and the arithmetic mean is accurate to 0.1 MPa [21]. Each half-prism was placed between two steel plates and loaded at a force rate of (2400 ± 200) N/s until failure. The compressive strength ${\;f\;}_{c}$ was determined by means of Equation (2) provided below:

(2) $$f_{c}\;=\;\frac{F_{c}}{A}$$

where $F_{c}$ is the maximal load (N), and A is the load-bearing area (40 × 40 mm^2^). For every mixture and every curing age, the mean values and standard deviations of the flexural and compressive strengths were documented. All testing equipment was calibrated regularly to ensure the load accuracy within ±1%.

We used Origin (2022) software to perform one-way ANOVA followed by Tukey post hoc tests to assess the significance of differences between groups [29]. All data are expressed as mean ± standard deviation (SD, n = 3), and *p* < 0.05 was defined as statistically significant.

#### 2.3.3. Drying Shrinkage Test Method

Pursuant to the national standard JGJ/T 70-2009 [26,30] Test Methods for Basic Properties of Construction Mortars, measurements of the drying shrinkage of cement mortar samples were carried out utilizing a BC-176 mortar shrinkage tester. A 4 h preconditioning treatment was applied to 160 mm × 40 mm × 40 mm cement mortar specimens (20 ± 2 °C, relative humidity 60 ± 5%), following which the initial length of the specimens was determined. The samples were then placed under natural curing conditions, and their lengths were measured at curing ages from 1 day to 28 days, respectively. The shrinkage rate was calculated based on the measured data. Three samples were examined simultaneously per group, with the drying shrinkage value defined as the arithmetic mean of the three measured results.

The shrinkage rate of each specimen can be calculated using [31] Equation (3):

(3) $$\varepsilon_{\mathrm{at}}\;=\;\frac{L_{0}-L_{t}}{L{\;-\;L}_{d}}$$

where $\varepsilon_{\mathrm{at}}$ is the natural drying shrinkage value at time t; *L*_0_ is the initial length of the sample (mm); *L* is the nominal length of the sample, i.e., 160 mm; *L_d_* is the sum of the embedded lengths of the two shrinkage heads in the mortar, i.e., 20 ± 2 mm; *L_t_* is the determined length of the sample at day t (mm).

#### 2.3.4. Mass Loss Rate Test Method

Mass evolution of the cement mortar specimens was recorded over 28 days with an electronic balance (2 kg, 0.01 g). Given the high humidity under standard curing conditions, which exerts a negligible effect on the volumetric water reduction ratio of the specimens, the present investigation was limited to assessing the impact of ambient curing conditions on the volumetric water reduction ratio of the specimens. Upon completion of molding, the samples underwent normal curing for 24 h before demoulding, and their initial mass $m_{0}$ was measured immediately afterwards. The mass $m_{t}$ of the specimens at curing ages from 1 day to 28 days was determined successively, and the mass loss rate was calculated using [32] Equation (4):

(4) $$\delta \;=\;\frac{m_{0}-m_{t}}{m_{0}}\;\times \;100\%$$

where δ is the mass loss of the samples at various curing ages, %; $m_{0}$ is the initial mass of the specimens immediately after demoulding, g; $m_{t}$ is the mass of the samples at various curing ages, g.

#### 2.3.5. EIS Tests

The prepared mixed paste was cast into a mold specially designed for AC impedance tests, and the paste was vibrated thoroughly using a vibrating table to minimize the generation of air bubbles. The mold dimensions were 40 mm × 40 mm × 70 mm. A piece of stainless-steel mesh (40 mm × 50 mm) was fixedly placed 10 mm away from the inner wall on each side of the mold. Subsequently, the samples were wrapped with polyethylene film to minimize moisture evaporation from both the surface and interior, and then cured in a standard chamber at 20 ± 2 °C and >95% relative humidity. AC impedance tests were conducted at curing ages of 1 h, 6 h, 12 h, 1 d and 3 d, respectively. The test parameters were set as follows: frequency range spanning 0.01 Hz to 1 MHz, high-frequency current range with a minimum of 2 mA, 10 mV AC signal amplitude, 10 data points within each frequency interval, and scanning mode for impedance versus frequency. During the test, alligator clips were used to connect the cables to the stainless-steel mesh to ensure reliable electrical contact.

**Figure 3:**
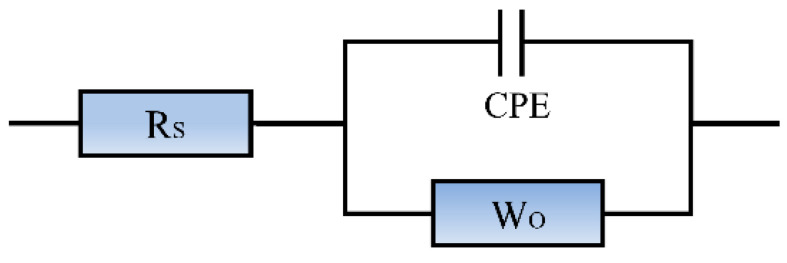
Equivalent circuit model.

In the analysis of AC impedance test results, the electrochemical reaction process inside the cementitious materials is usually fitted by establishing an equivalent circuit model. This method characterizes the evolution law of parameters corresponding to various physical processes under the action of sinusoidal alternating current through a model composed of series and parallel connections of different circuit components. In the study of cementitious material hydration, the core of AC impedance technology lies in analyzing the variation characteristics of each component parameter of the equivalent circuit with the progress of the hydration reaction [33]. In this study, the R_S_ (CPE (R_ct_//W_o_) equivalent circuit model presented in Figure 3 was utilized for fitting the EIS data. Among them, R_S_ represents the solution resistance of mortar pores; at the mortar–electrolyte interface, the non-ideal double-layer behavior is characterized by the Constant Phase Element (CPE); the charge transfer resistance in cementitious materials is denoted as R_ct_; and W_o_ corresponds to the Warburg impedance caused by the unrestricted diffusion of ions inside the mortar on the electrode surface. In the Nyquist plot, the vertical and horizontal axes represent the imaginary component and real component of impedance, respectively, and the test frequency reduces progressively along with the rise in the horizontal coordinate value. In the Nyquist plot, the diameter of the high-frequency semicircle along the real axis corresponds to the charge transfer resistance R_ct_. The transition point between the high-frequency semicircle and the low-frequency linear tail occurs where the imaginary component of the impedance approaches zero. In this test, the high-frequency semicircle is mainly distributed in the range of 10–10^6^ Hz, associated with the electrochemical reaction at the liquid-solid interface within the cement-based matrix, while the low-frequency region of 0.01–10 Hz reflects the diffusion-controlled process between the stainless-steel working electrode and the cementitious matrix. The impedance spectra of all cement mortar specimens exhibit similar overall characteristics, i.e., a mildly depressed semicircular feature in the high-to-medium-frequency domain and a slanted linear segment with a slope different from 45° in the low-frequency region. This characteristic originates from the dispersion effect at the interface formed between the hardened cementitious material and the electrode, which makes the ideal double-layer capacitance deviate from ideal behavior and thus is described by the CPE in the equivalent circuit.

#### 2.3.6. Electrical Resistivity Test Method

**Figure 4:**
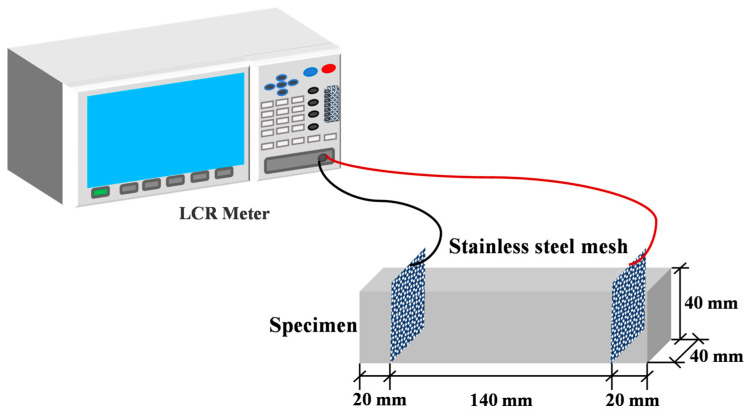
Two-electrode method-based schematic for mortar specimen ER measurement.

The electrical resistivity (ER) values of three samples were determined by a two-electrode configuration at curing ages from 1 day to 28 days. The electrical resistivity ρ was calculated from the volume resistance, which was determined via the embedded two-electrode method with stainless-steel meshes serving as electrodes. The test setup is schematically illustrated in Figure 4, and a digital LCR bridge was adopted for the measurement. Conductive stainless-steel mesh was chosen as the electrode material due to its good compatibility with the matrix, excellent durability, low polarization, and low contact resistance [34]. In the fabrication process of cement mortar prisms measuring 40 mm × 40 mm × 160 mm, two conductive stainless-steel meshes (40 mm × 50 mm) were embedded along the length of each specimen, with a spacing distance of 120 mm between them. To eliminate the polarization effect, alternating current with a frequency of 1 kHz was applied, and the duration of each test was less than 1 min. Under each test condition, three replicate specimens were tested simultaneously, with the average of the obtained results reported as the final value.

The ER was determined using Equation (5):

(5) $$\;\rho \;=\;R\frac{A}{L}$$

where *ρ* is the ER of the tested specimen (Ω·m); *R* is the measured resistance of the specimen at the test point (Ω); *A* is the area of the embedded electrodes (m^2^); L is the center-to-center distance between the two electrodes (m).

#### 2.3.7. Chloride Electric Flux and Diffusion Coefficient

**Figure 5:**
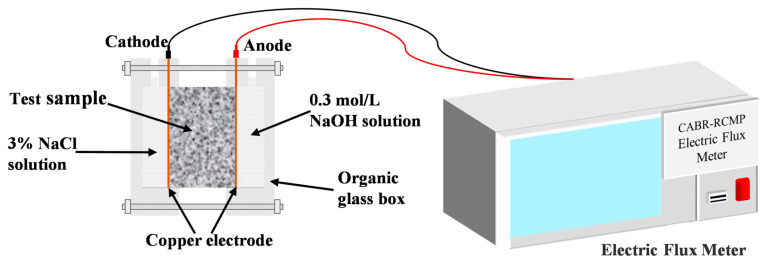
Schematic illustration of the electrical flux technique for mortar specimens.

The chloride electric flux (CEF) test was conducted on concrete in compliance with GB/T 50082-2024 [12]. A concrete CEF tester was adopted as the test apparatus, and the test setup is shown in Figure 5. The samples had a cylindrical shape with a 50 mm height and a 100 mm diameter. After the specimens were subjected to vacuum saturation in a saturation apparatus, they were taken out of the water and installed in the test cell. The test chamber attached to the negative electrode was filled with a 3.0% NaCl solution, whereas the chamber connected to the positive electrode contained a 0.3 mol/L NaOH solution. The power supply was activated, a constant diffusion coefficient (DC) voltage of 60 V was imposed, and the initial current value $I_{0}$ was documented. The current value was measured at 30 min intervals until the power was supplied for 6 h.

The total coulomb electric flux (TCEF) of each sample could be computed via the simplified Equation (6) provided below, with the result accurate to 0.01 C:

(6) $$Q\;=\;900(I_{0}\;+\;2I_{60}\;+\;2I_{90}\;+\;2I_{120}\;+\;2I_{150}\;+\;2I_{180}\;+\;2I_{210}\;+\;2I_{240}\;+\;2I_{270}\;+\;2I_{300}\;+\;2I_{330}\;+\;2I_{360})$$

where Q is TCEF (C); $I_{0}$ is initial current (A); $I_{t}$ is current at time t (A).

The chloride ion diffusion coefficient (*D*) could be determined using Equation (7) [31]:

(7) $$D\;=\;(0.00492\;\times \;Q\;+\;2.582)\;\times \;10^{-12}\;$$

where D is chloride ion DC, measured in m^2^/s.

#### 2.3.8. Carbonation Depth Test Method

Rapid carbonation assessment of concrete was performed in compliance with GB/T 50082-2024 [35]. After being subjected to standard curing for 26 days, the specimens were dried in an oven at 60 °C for a duration of 48 h. Molten paraffin wax was applied to seal the side and bottom surfaces of the samples, with only the opposing faces retained in an exposed state. Parallel reference lines were scribed at 10 mm spacing along the unsealed surfaces, acting as benchmarks for subsequent carbonation depth (CD) determination. An accelerated carbonation experiment was carried out in a TH-B carbonation chamber, where the CO_2_ concentration, relative humidity, and temperature were controlled at 20 ± 0.5%, 70 ± 5%, and 20 ± 2 °C, respectively. Specimens were sampled and tested after 1, 3, 7, 14, and 28 days of curing. After each testing interval, the samples were split, and residual powder on the fractured surfaces was removed. The surfaces were sprayed with a 1% phenolphthalein solution, and the carbonation depth was determined at the marked locations after 30 s using a steel ruler. The final CD was determined as the mean value of the measured data points, which is a key index for evaluating carbonation. According to GB/T 50082-2024 the CD was determined using Equation (8):

(8) $$d_{t}\;=\;\frac{1}{n}\sum_{i=1}^{n}d_{i}$$

where $d_{t}$ is the average CD after time *t* (mm); $d_{i}$ is the CD at every point (mm); n is all points.

#### 2.3.9. Microstructure

Typical freshly fractured samples after the compressive strength testing were sampled and submerged in anhydrous ethanol, which was replaced daily to prevent further hydration reactions. The samples were then dehydrated to constant weight in a 40 °C vacuum oven. The microstructure of SAC in the samples was analyzed using a field-emission environmental scanning electron microscope (SEM). This instrument was fabricated by FEI Company, Hillsboro, OR, USA, with the model number Quanta FEG 250. A secondary electron resolution of less than 1.0 nm under high-vacuum mode, a secondary electron resolution of less than 1.4 nm under low-vacuum mode, and an energy spectrum resolution of 127 eV.

## 3. Results and Discussion

### 3.1. Workability

#### 3.1.1. Fluidity

**Figure 6:**
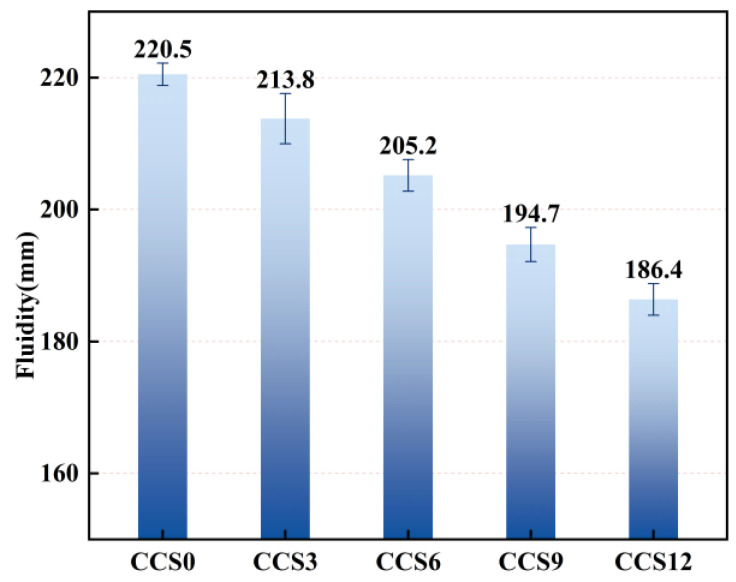
Fluidity of SAC systems with different CCS doping levels.

The error bars in Figure 6 represent the standard deviation of three independent measurements, indicating acceptable repeatability of the fluidity tests. CCS0 exhibited a maximum fluidity of 220.5 mm, followed by CCS3 and CCS6 with fluidity values of 213.8 mm and 205.2 mm, respectively; CCS9 and CCS12 showed the minimum fluidity, corresponding to 194.7 mm and 186.4 mm. Compared with CCS0, the fluidity of CCS3 decreased by 3.04%, that of CCS6 by 6.94%, that of CCS9 by 11.70%, and that of CCS12 by 15.46%, suggesting that the incorporation of CCS markedly reduces the flowability of fresh mortar. As the CCS content was raised from 0% to 12%, a continuous reduction in the fluidity of SAC mortar was observed, reflecting the inherently high fluidity of pure SAC mortar.

In SAC systems incorporating CCS, the fluidity decreases continuously with increasing CCS dosage due to CCS’s strong water absorption capacity and high specific surface area. CCS’s physical morphology is primarily characterized by porous, angular particles. This physical appearance not only enhances CCS’s ability to physically adsorb free water but also increases the internal friction between CCS and SAC particles, thereby causing a continuous decline in the amount of free water responsible for lubricating the SAC mortar matrix [36]. The incorporation of CCS also results in the presence of unhydrated free CaO and active silica–alumina components in the SAC system. These components are able to participate in the initial hydration processes of the SAC system. During the reaction, these materials continuously consume additional free water and form an early hydration film on the particle surface, further restricting the movement of internal material particles and thus reducing the fluidity of the SAC system [37].

When 6% CCS is added to the SAC system, its fluidity decreases by 15.3 mm compared to the sample without CCS, a reduction of 6.94%. At this dosage, the fine particle filling effect of CCS improves particle packing and reduces pore volume, thus offsetting, to some extent, the water uptake induced by CCS and resulting in only a slight decrease in the overall flowability of the SAC system. When the CCS content in the SAC system exceeds 9%, its overall fluidity decreases by 25.8 mm compared to the sample without CCS, a reduction of 11.70%. At this dosage, the water absorption effect of CCS, particle friction, and decreased mortar continuity become the dominant factors in the SAC system, leading to increased cohesion and decreased fluidity within the material. This indicates that high dosages of mineral admixtures significantly reduce the workability of cement mortar [38].

CCS, as an admixture, can improve the physical pore filling effect of the SAC system, so as to ensure its later mechanical properties and dense microstructure [39]. From a chemical interaction perspective, the improvement in workability observed with CCS incorporation can be attributed to its calcium-rich composition. Calcium-bearing phases such as Ca(OH)_2_ and hydration products including C–S–H are capable of forming strong hydrogen bonds with water molecules, which enhances water adsorption and provides a lubrication effect among solid particles. This interaction facilitates particle dispersion and improves flowability. In contrast, systems enriched in SiO_2_ exhibit weaker hydrogen bonding with water, leading to reduced water retention and consequently lower workability. Therefore, the chemical composition of CCS plays a crucial role in regulating the fresh-state behavior of SAC-based mortars [40]. In the actual engineering construction process, the fluidity required for pouring should be met without significantly reducing the early hydration reaction rate, so as to ensure a reasonable balance between its fluidity and later performance, thereby ensuring the development of overall performance [13].

#### 3.1.2. Setting Time

**Figure 7:**
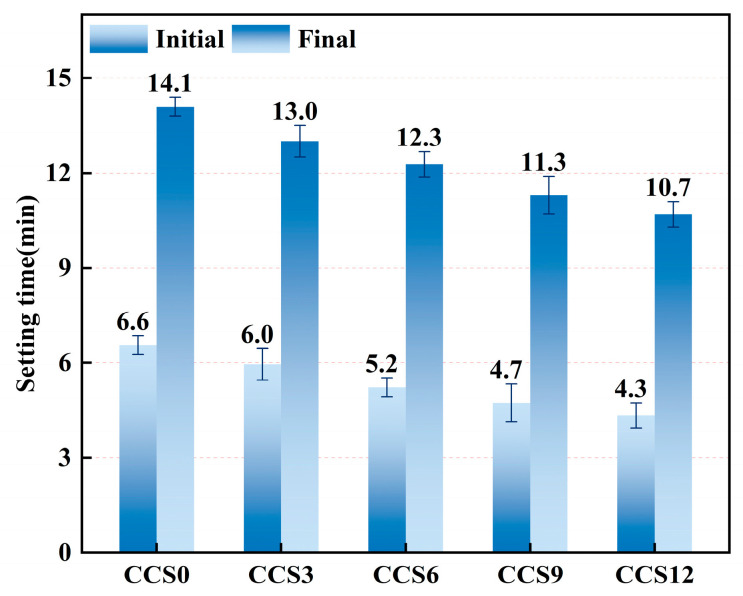
Setting time of SAC systems with different CCS doping levels.

The relatively small standard deviation in Figure 7 indicates that the setting time measurement has good repeatability and experimental reliability, the setting times of SAC mortars with varying CCS dosages are compared. For the control specimen CCS0 (0% CCS), the initial and final times were measured as 6.6 min and 14.1 min. Relative to the control specimen CCS0, the initial times of CCS3, CCS6, CCS9, and CCS12 with CCS dosages of 3%, 6%, 9%, and 12% decreased by 9.26%, 20.37%, 27.78%, and 33.93%, respectively, and the final setting times reduced by 7.76%, 12.93%, 19.83%, and 24.14%, respectively. As the CCS dosage increased, both initial and final setting times were reduced, suggesting that CCS incorporation accelerates the early hydration process of the SAC system.

As the CCS dosage rose from 0% to 12%, the initial setting time shortened from 6.6 min to 4.3 min, and the final setting time decreased correspondingly from 14.1 min to 10.7 min. This accelerating effect of CCS on the early hydration of the SAC system mainly arises from the high alkalinity and small particle size of CCS, which promotes the dissolution reaction of silicates and aluminates within the SAC system, thereby enhancing the generation and maturation of hydration products in the CCS-SAC reaction process. CCS rich in Ca(OH)_2_ residue can act as an effective alkaline activator, shortening the induction period of the SAC system and increasing its reactivity [38].

As the CCS dosage increased, the setting time of the SAC system progressively decreased, indicating that incorporating CCS provides abundant nucleation sites and accelerates the generation of Aft and C-S-H in the SAC system. The fine CaCO_3_ and Ca(OH)_2_ particles inside CCS can enhance the heterogeneous nucleation and hydration exothermic process of the initial reaction phase of the SAC system [41]. When the content of CCS as an admixture in the SAC system is 3%, the hydration acceleration effect is relatively slow, indicating that there is a certain balance between the alkalinity of CCS itself and the dilution of the SAC system, making the hydration acceleration relatively mild. Due to the increase in OH^−^ concentration provided by CCS, the early hydration process of C_3_A and C_4_AF phases in the SAC system is moderately accelerated, consequently stimulating the generation of AFt and C-A-H. When the CCS content in the SAC system reaches 6–9%, the setting time of the SAC system continues to shorten, but the rate of shortening of the setting time slows down, indicating that the hydration acceleration effect of the SAC system under this CCS doping is gradually approaching saturation. The incorporation of CCS ensures that there is enough Ca(OH)_2_ in the SAC aiding the accelerated formation AFt [42]. The particle filling influence of CCS also refines the pore structure of the SAC system, improves the continuity of the solid phase, and thus promotes faster condensation of hydration products [39].

When the CCS content in the SAC system reaches 12%, its setting time continues to shorten, but the shortening is less than that of 6–9% CCS content. This may be because the excessive CCS in the SAC system replaces the SAC, resulting in a decrease in the effective content of tricalcium silicate (C_3_S) and C_3_A, thereby slowing down the later hydration reaction. Excessive OH^−^ concentration may cause premature crystallization of Ca(OH)_2_ on the surface of particles in the SAC system to hinder internal ion diffusion, thereby slightly offsetting the effect of accelerating setting [43]. When the CCS content is 6–9%, the interactive influence of chemical activation, particle filling and alkali stimulation in the SAC system is enhanced to a certain extent, forming an internal equilibrium state; when the CCS content exceeds this range, the dilution effect in the SAC system dominates, and the control effect on setting time is poor.

### 3.2. Mechanical Properties

#### 3.2.1. Flexural Strength

**Figure 8:**
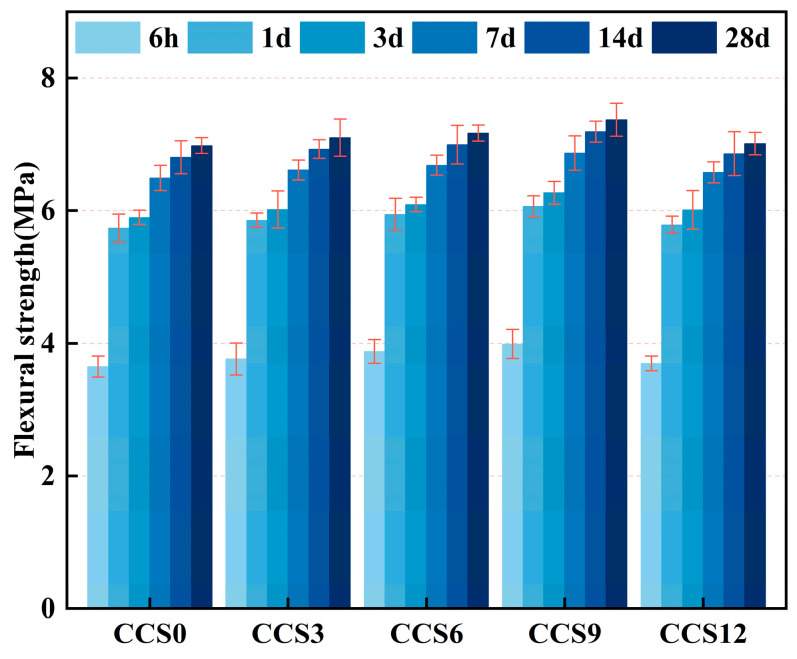
Influence of CCS dosage on the flexural strength of the SAC system.

Figure 8 depicts the development of flexural strength in cement mortar specimens with different CCS contents during curing periods of 6 h, 1 day, 3 days, 7 days, 14 days, and 28 days. The experimental groups at different curing ages all exhibited relatively small standard deviations, indicating that the flexural strength test has acceptable repeatability. Compared with the CCS0 specimen, the flexural strength values at various curing periods were 6 h (3.65 MPa), 1 d (5.74 MPa), 3 d (5.90 MPa), 7 d (6.49 MPa), 14 d (6.80 MPa), and 28 d (7.01 MPa), indicating that the flexural strength added gradually with the extension of curing periods. The flexural strength at 6 h reached 52.14% of that at 28 d, 81.97% at 1 d, 84.26% at 3 d, 92.75% at 7 d, and 97.14% at 14 d. The strength increased rapidly within the first day, which aligns with the inherent characteristic of SAC featuring fast early strength development [20].

At the curing period of 6 h, the flexural strength values of different cement mortar specimens were: 3.65 MPa for CCS0, 3.76 MPa for CCS3, 3.88 MPa for CCS6, 3.99 MPa for CCS9, and 3.70 MPa for CCS12. As the CCS content increased gradually, flexural strength exhibited a pronounced increase followed by a decline, attaining its maximum at 9% CCS, and falling as the CCS content rose to 12%. This trend indicates that an appropriate dosage of CCS can promote the early reactivity and structural densification of the SAC system, whereas excessive incorporation will impair the overall mechanical properties. Compared with CCS0, the flexural strength of CCS3 increased by 3.10%, that of CCS6 by 6.19%, that of CCS9 by 9.26%, and that of CCS12 by 1.32%. At this age, the main hydration product of the mortar specimens with CCS addition was ettringite (AFt), accompanied by the production of some monosulfate (AFm) phase, along with a small amount of calcium carboaluminate possibly formed. In comparison with the specimens without CCS, the AFt content increased significantly, the proportion of AFm phase rose, and the overall hydration process was accelerated. The introduction of CCS furnished extra Ca^2+^ and OH^−^ ions, which greatly increased the liquid phase alkalinity and thereby accelerated the hydration of anhydrous calcium sulfoaluminate (C_4_A_3_Ŝ) [43]. The filling effect of CCS can also accelerate the early formation of the structural framework in the SAC. While the CCS amount in the SAC reaches 12%, the 6 h flexural strength shows a slight decrease. This may indicate the presence of excessive inert particles within the CCS, reducing the effective cement content in the SAC system and resulting in insufficient early structural density [44].

At the curing period of 1 d, the measured flexural strength of every specimen was: 5.74 MPa for CCS0, 5.86 MPa for CCS3, 5.95 MPa for CCS6, 6.06 MPa for CCS9, and 5.79 MPa for CCS12. Compared with CCS0, the flexural strength of CCS3 raised by 0.12 MPa, CCS6 by 0.21 MPa, CCS9 by 0.32 MPa, and CCS12 by 0.05 MPa, with the most significant improvement observed at a CCS content of 9%. At this age, C_4_A_3_Ŝ in the SAC continued to react vigorously with Ca(OH)_2_ and CaSO_4_·2H_2_O provided by CCS to generate AFt; meanwhile, CCS accelerated the hydration of C_2_S to form C-S-H gel [45].

With the increase in curing age, the flexural strength values at 3 d were: 5.90 MPa for CCS0, 6.02 MPa for CCS3, 6.09 MPa for CCS6, 6.27 MPa for CCS9, and 6.01 MPa for CCS12. Compared with the flexural strength at 1 d, the values increased by 0.16 MPa for CCS0, 0.16 MPa for CCS3, 0.14 MPa for CCS6, 0.21 MPa for CCS9, and 0.22 MPa for CCS12. The improvement was marginal relative to that at 1 d, primarily owing to the following reasons: the hydration rate of C_4_A_3_Ŝ decreased significantly (with approximately 60–70% of the mineral content consumed), and the AFt production tended to be saturated; gypsum was gradually depleted, leading to insufficient SO_4_^2−^ in some areas and thus the evolution of a small amount of AFt to AFm phase; AH_3_ further reacted with Ca^2+^ and SiO_4_^4−^ to form C-(A)-S-H gel, which enhanced the cohesion between hydration products.

At the curing periods of 7 d, 14 d and 28 d, the samples of the flexural strength increased gradually, peaking at 28 d. For CCS0, the flexural strength was 6.49 MPa (7 d), 6.81 MPa (14 d), and 6.98 MPa (28 d). The strength at 14 d was 0.32 MPa higher than that at 7 d, and the strength at 28 d was 0.17 MPa higher than that at 14 d, with the rate of strength increment slowing down. This demonstrates that the strength development of SAC is largely concentrated within the first 3 d. In 28 d, the flexural values were: 6.98 MPa for CCS0, 7.10 MPa for CCS3, 7.17 MPa for CCS6, 7.37 MPa for CCS9, and 7.01 MPa for CCS12. The optimal CCS content for achieving the ultimate flexural strength of the mortar specimens was 9%, corresponding to a 5.59% increase compared with CCS0 without CCS addition. At this age, a substantial quantity of C-S-H gel was generated in the system, and AFt remained basically stable. The incorporation of 9% CCS promoted the filling, cross-linking and spatial framework optimization of the gel phase, thereby enhancing the long-term strength accordingly [39].

#### 3.2.2. Compressive Strength

**Figure 9:**
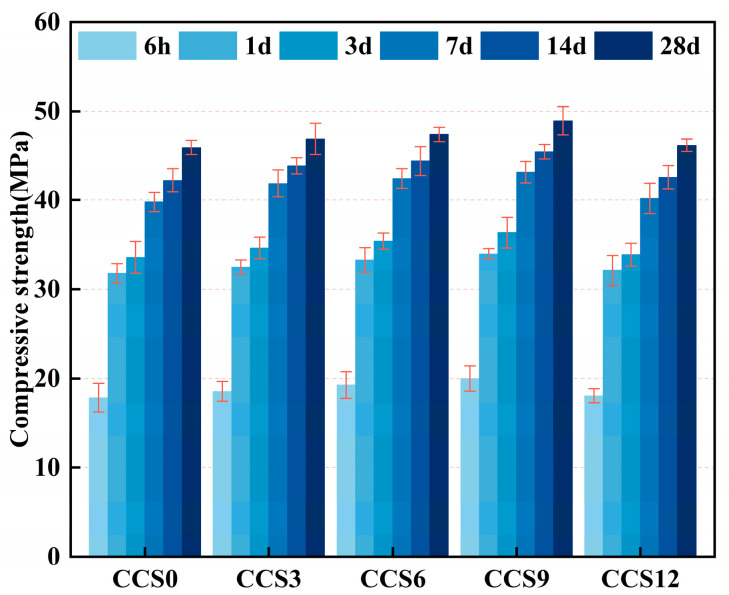
Variation in compressive strength of SAC system with different CCS doping levels.

As shown in Figure 9, the experimental groups at different ages all showed relatively small standard deviations, indicating that the compressive strength test has acceptable repeatability. The compressive strength values of CCS0 at various curing periods were recorded as follows: 6 h (17.84 MPa), 1 d (31.81 MPa), 3 d (33.60 MPa), 7 d (39.80 MPa), 14 d (42.25 MPa), and 28 d (45.91 MPa). The rapid growth of compressive strength mainly occurred within 6 h and 1 d. The compressive strength at 6 h reached 38.86% of that at 28 d, and the strength at 1 d accounted for 69.29% of the 28 d strength. When the curing period reached 3 d, the compressive strength corresponded to 73.19% of its 28-day value, which reflects the typical high early-age strength and fast-curing characteristics of the SAC system. In comparison with the 28 d compressive strength, the strength at 7 d reached 86.69%, while that at 14 d reached 93.37%, indicating that the compressive strength of the mortar specimens raised continuously with the expansion of curing period, but the growth rate gradually decreased.

At a curing period of 6 h, the strengths of the SAC systems with CCS content of 0%, 3%, 6%, 9%, and 12% were 17.84 MPa, 18.56 MPa, 19.28 MPa, 19.99 MPa, and 18.06 MPa, respectively. The highest strength was found in the SAC system with 9% CCS content, which was 12.05% higher than the SAC system without CCS. The strength trend of the SAC systems at 6 h was similar to that of the flexural strength, indicating that the mechanical properties were relatively optimal with 9% CCS content. In 1 day, the compressive of the mortar samples of all SAC systems was significantly greater than that at 6 h. The compressive strengths of the CCS content from 0% to 12% were 31.81 MPa, 32.52 MPa, 33.31 MPa, 34.02 MPa, and 32.13 MPa, respectively, all exceeding 30 MPa. The strength of SAC mortar specimens with various CCS contents all showed varying degrees of improvement, with the SAC system with 9% CCS content exhibiting the highest compressive strength. C_4_A_3_Ŝ in the SAC system possesses rapid hydration reaction capabilities; it can rapidly hydrate with CCS-derived SO_4_^2−^ and Ca^2+^, generating a high quantity of AFt and promoting the prompt generation of the overall structure of the SAC system [45].

At 3 days of age, the strength of SAC mortar samples with CCS content ranging from 3% to 12% was exceeding that of the control sample without CCS. The compressive followed the order CCS9 > CCS6 ≈ CCS3 > CCS0. The strength of CCS9 was 36.40 MPa, which was 2.80 MPa higher than that of CCS0. After 7, 14, and 28 days of curing, the strength of all mortar specimens continued to increase and peaked at 28 d, with the final values being 45.91 MPa for CCS0, 46.86 MPa for CCS3, 47.37 MPa for CCS6, 48.91 MPa for CCS9, and 46.16 MPa for CCS12. CCS9 demonstrated the highest compressive strength, primarily ascribed to the synergistic effect between SAC hydration and CCS-induced secondary hydration reactions. The presence of CCS accelerated the early transformation of AFt to the stable monosulfate (AFm) phase and enhanced the continuous generation of C-(A)-S-H gel, thereby significantly increasing the microstructural densification. Excessive CCS fine particles would increase the water demand of the entire system, affect the operative W/B ratio, and consequently reduce the strength. The incorporation of CCS enables the SAC system to undergo continuous hydration reactions, stimulating the ongoing development of AFm, thus ensuring the long-term reinforcing effect of the SAC system. The secondary hydration effect of CCS and SAC fills the capillary pores, improves the internal pore configuration of the SAC system mortar samples, and reduces the overall porosity [39].

Incorporating CCS into the SAC system can achieve a certain strength improvement at all ages, and the mechanical strength improvement of the SAC system varies with different CCS dosages. The mechanical strength improvement is greatest when the CCS dosage is 9%, while the strength of the SAC system is lower than that of the sample with 9% CCS dosage when the CCS dosage reaches 12%. This phenomenon was mainly ascribed to the chemical reactivity of CCS: CCS contains Ca(OH)_2_ and residual CaSO_3_/CaSO_4_, which can promote secondary hydration reactions with the aluminate phases in SAC to generate more AFt, thus forming a denser microstructure. In contrast, excessive CCS incorporation, due to its high sulfate content and unreacted CaSO_3_, leads to the formation of early-stage unstable AFt with large volume, which delays structural densification. Additionally, the pore coarsening caused by the decomposition of unstable AFt and incomplete hydration due to unbalanced sulfate content also contribute to strength reduction.

### 3.3. Characterization of Hydration Degree

#### 3.3.1. Drying Shrinkage

**Figure 10:**
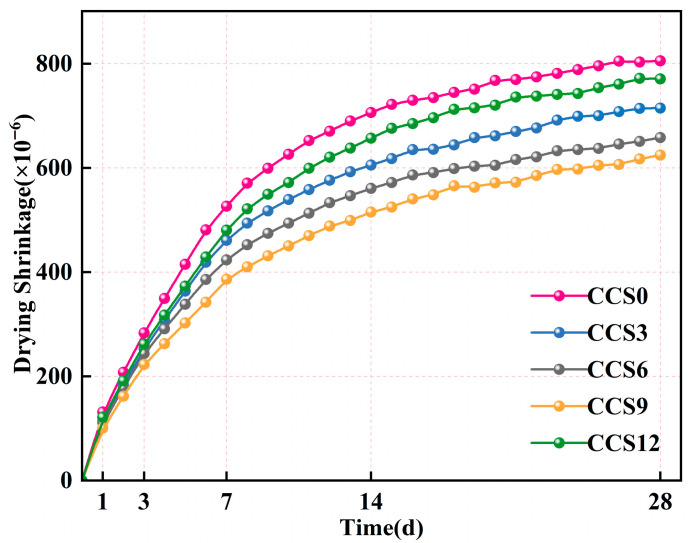
Drying shrinkage strain evolution in multiple mortar specimen groups.

Figure 10 depicts the evolution of drying shrinkage of SAC mortar specimens with different CCS contents over a 28-day period. It is evident from the experimental results that the drying shrinkage exhibited a correlation with CCS content. CCS0 without CCS addition exhibited the highest shrinkage rate throughout the curing period from 1 d to 28 d. As the CCS content raised from 0% to 9%, the shrinkage rate gradually decreased, while a marginal rise appeared at a CCS content of 12%. This indicates that adding an appropriate amount of CCS to the SAC system can optimize the overall pore structure layout and reduce the overall internal structure shrinkage. When the CCS content is too high, there will be unhydrated parts in the SAC system, which will lead to the formation of more fine pores and increase capillary stress and overall shrinkage [46].

After 1 day of curing, the SAC system exhibited a drying shrinkage of about 0.1‰ for all CCS dosages, suggesting that the early shrinkage was scarcely affected by the amount of CCS. This was because the mortar still remained in a state with a substantial quantity of free water, and the internal relative humidity was close to saturation, thus inhibiting the build-up of capillary stress. The rapid build-up of AFt acicular crystals in the SAC matrix at the very early stage exerted a stiffness-enhancing effect, minimizing transient deformation. Therefore, the shrinkage at 1 d was mainly affected by the redistribution of initial water rather than changes in pore structure.

At 3 d, differences in drying shrinkage among different specimens began to emerge. Compared with CCS0, the drying shrinkage of CCS3 decreased by 10.99%, that of CCS6 by 14.13%, that of CCS9 by 21.42%, and that of CCS12 by 7.66%. The reduction in shrinkage rate induced by CCS incorporation was mainly attributed to two mechanisms. First, CCS participated in the secondary reactions of residual CaO/Ca(OH)_2_ and aluminates to generate additional AFm/AFt phases, forming a denser skeleton. The improved pore connectivity reduced the effective capillary tension, thereby inhibiting shrinkage [39]. Second, CCS particles retained part of the mixing water and gradually released it during hydration, slowing down the decrease in internal relative humidity and thus controlling early-stage shrinkage. This phenomenon is consistent with the moisture diffusion-controlled shrinkage mechanism of the rapid-hardening calcium sulfoaluminate system: the loss of early-stage physically bound water and rapid formation of AFt lead to the rapid development of capillary tension, while later-stage shrinkage is mainly controlled by the slow evaporation of water in fine gel pores [39].

The drying shrinkage of CCS0 at 7 d reached 65.40% of that at 28 d. For the other groups, the ratios of 7 d drying shrinkage to 28 d drying shrinkage were 64.55% for CCS3, 64.34% for CCS6, 61.87% for CCS9, and 62.38% for CCS12, respectively. All groups exhibited a high magnitude of shrinkage within the initial 7 d, following which the rate of shrinkage growth progressively decreased and eventually stabilized in the later stage. In 14 day and 28 day, the increase in drying shrinkage of all mortar specimens continued to slow down, and all curves approached asymptotic values.

At 28 d, compared with CCS0, the drying shrinkage of CCS3 decreased by 11.29%, that of CCS6 by 18.26%, that of CCS9 by 22.47%, and that of CCS12 by 4.34%. The reduction magnitude of drying shrinkage for CCS12 was lower than that for CCS6 and CCS9, which can be explained as follows: excessive sulfate supply promotes the formation of acicular AFt in confined spaces, which may induce microcracks and increase later-stage shrinkage [47]. The high content of fine particles in CCS increases the number of gel pores (<10 nm), facilitating long-term moisture diffusion and enhancing capillary stress.

#### 3.3.2. Mass Loss Rate

**Figure 11:**
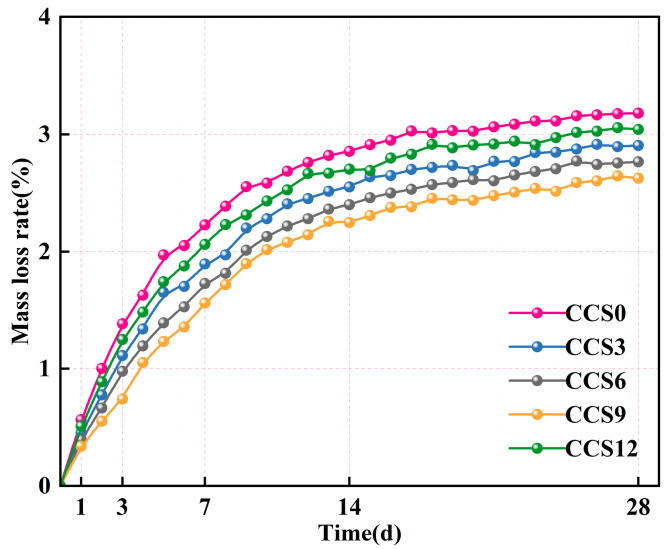
Mass loss rate progression curves for various mortar sample groups.

Figure 11 shows the mass loss rate (MLR) of mortar specimens with different CCS dosages at various curing ages. The MLR of CCS0 at various periods were as follows: 0.566% (1 d), 1.385% (3 d), 2.228% (7 d), 2.854% (14 d), and 3.182% (28 d). The MLR increased continuously with the prolongation of curing period, indicating the gradual evaporation of free water and continuous hydration of the binder phase. Following 7 days of curing period, the growth rate of mass loss rate slowed down for all specimens, suggesting that the consumption of pore water and moisture exchange tended to reach a balance in the later stage.

In the initial period (1–3 d), all mortar specimens exhibited rapid mass loss, which was caused by the evaporation of physically adsorbed water and heat release from initial hydration. The CCS amount added 0–9%, and the SAC system reduced continuously. This indicates that there are fine CaCO_3_ and Ca(OH)_2_ particles in CCS, which can fill the capillary pores and delay the surface drying [48]. When the age is 7 d, the rise in CCS content has a more significant influence on the mass loss rate of the SAC system. The SAC system without CCS has the maximum mass loss, while the SAC system with 9% CCS has a mass loss of 1.560%, which is 0.668% less than the mass loss rate without CCS. The mass loss of the SAC system with 12% CCS is higher than that with 9% CCS content. This may be because the excessive CCS consumes the water in the SAC system, resulting in incomplete hydration. Adding 9% CCS can effectively reduce the overall water loss of the SAC system by improving particle packing and maintaining normal hydration [15].

At the curing period of 14 d, the MLRs of the mortar specimens were as follows: 2.904% for CCS0, 2.904% for CCS3, 2.765% for CCS6, 2.625% for CCS9, and 3.043% for CCS12. Compared with the MLR at 7 d, the increase in MLR tended to stabilize, which was mainly because the long-term hydration of aluminate and silicate phases consumed the available Ca(OH)_2_, while the C-S-H gel and AFt formed during the latter period stage filled the residual voids [47].

At 28 d, the MLR of the mortar specimens were as follows: 2.904% for CCS3, 2.765% for CCS6, 2.625% for CCS9, and 3.043% for CCS12. Compared with CCS0, the addition of CCS reduced the mass loss rate, and this trend was maintained as the replacement rate increased up to 9%. This was attributed to the filling and pozzolanic effects of CCS rich in Ca(OH)_2_, which formed a denser microstructure and reduced the evaporation rate of the internal pore network [38]. The MLR of CCS12 was greater than that of CCS9, which may be due to the reduced proportion of cement caused by excessive CCS incorporation; the cement dilution resulted in a lower degree of hydration of the overall system.

### 3.4. Characterization of Hydration Characteristics

#### 3.4.1. EIS

**Figure 12:**
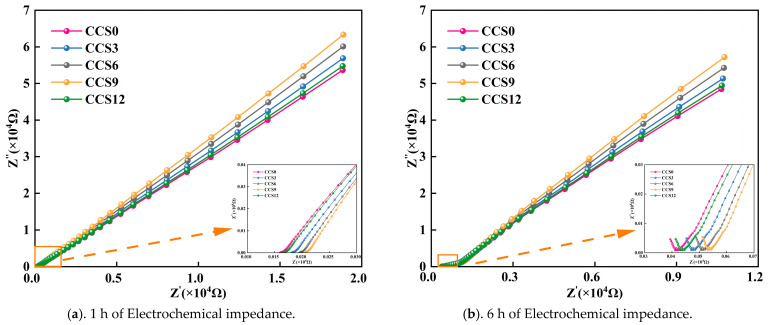

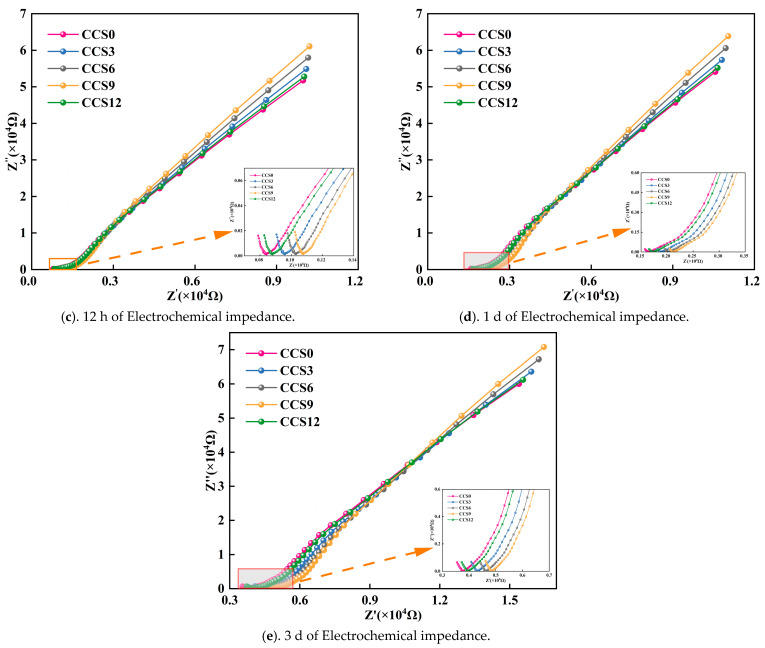
Electrochemical impedance changes in SAC systems with different CCS doping levels.

Figure 12a–e present the Nyquist plots of SAC mortar specimens with various CCS contents at the early hydration periods of 1 h, 6 h, 12 h, 1 d and 3 d. For CCS0 in Figure 12a–e, as the hydration period increased, the impedance value R_ct_ at the junction between the high-frequency semicircle and the low-frequency line exhibited a progressive increase. The real-part impedance values of CCS0 at hydration ages of 1 h, 6 h, 12 h, 1 d and 3 d were 157 Ω, 394 Ω, 787 Ω, 1575 Ω and 3551 Ω, correspondingly. This is manifested in the plots as a continuous rightward shift in R_ct_ (the resistance associated with the internal charge transfer reaction in the cementitious system), and the same trend was observed for CCS3, CCS6, CCS9 and CCS12.

As depicted in Figure 12a–e, the impedance of SAC mortar samples with different CCS contents increased systematically, reflecting the gradual refinement and increased discontinuity of the pore structure with the advancement of hydration reactions. At the early stage of 1–12 h, the impedance response was dominated by the conductive pore solution, resulting in relatively low R_ct_ values. When the hydration reaction proceeded to 1 d and 3 d, the generation and buildup of hydration products, especially AFt and C-A-S-H gel in the SAC system, effectively reduced pore connectivity and restricted ion transport, thus leading to higher impedance values. In addition, the gradient of the low-frequency linear portion increased with the prolongation of curing period, indicating enhanced diffusion resistance. This phenomenon is usually attributed to the gradual transition of the pore network from a capillary-dominated type to a gel-pore-dominated type, which significantly hinders long-range ion migration. Similar impedance evolution has also been observed in OPC and SAC systems and is considered a reliable electrochemical indicator of microstructural densification [49].

At each hydration age, the mortar specimens with different CCS contents exhibited significant differences in impedance response, indicating that the introduction of CCS had a remarkable influence on the early hydration kinetics and pore structure development of SAC mortar. At 1 h, all mortar specimens showed relatively low impedance values, indicating that the electrochemical response was dominated by pore solution conductivity. The R_ct_ values of CCS-modified samples were already slightly higher than that of CCS0, with 157 Ω for CCS0, 181 Ω for CCS3, 195 Ω for CCS6, 205 Ω for CCS9 and 165 Ω for CCS12. The results show that when the CCS doping content is less than 9%, the early impedance value of the SAC system at 1 h continuously is elevated with the rise in CCS doping content. This may be due to the high specific surface area and small particle size of CCS, which enhances the physical adsorption of unlimited liquid in the SAC system and curtails the effective migration rate of ions in the pore solution [12,13]. As the hydration reaction proceeds, the difference in impedance value R_ct_ of the SAC system with different CCS doping contents becomes more and more obvious. At 12 h, when the CCS amount in the SAC improves (0–12%), the impedance values R_ct_ are 787 Ω, 905 Ω, 976 Ω, 1024 Ω and 827 Ω, respectively. The impedance value of the SAC system with CCS doping is higher than that without CCS doping, indicating that the alkalinity of CCS and the CaO content are conducive to accelerating the ongoing hydration reaction of the SAC system, and the released Ca^2+^ ions also accelerate the formation of AFt, thereby filling the pores of the SAC system in the early stage and forming a microstructure with higher impedance value. The SAC system with 12% CCS has a lower impedance than the SAC system with 9% CCS. This may be because the excessive CCS in the SAC system leads to uneven early hydration and reduced connectivity of local pores [48].

At 1 day and 3 days, the impedance spectra of the SAC system with different amounts of CCS were separated into different clusters. At 3 days, the impedance values of the SAC system with CCS doping increasing from 0% to 12% were 3551 Ω, 4084 Ω, 4403 Ω, 4616 Ω, and 3729 Ω, respectively. The SAC system with 9% CCS doping had the highest impedance value, indicating that its pore structure was finer than other SAC systems and that it had greater resistance to ion conduction. This indicates that there is an optimal synergistic influence between the hydration products of the SAC system and the CCS-derived Ca(OH)_2_, which enhances the secondary reactions within the SAC system and improves the densification of the microstructure. The SAC system with 12% CCS doping had a higher impedance value than the SAC system without CCS, which may be associated with the local agglomeration of slag granule and the imbalance between the hydration rate and pore filling efficiency when excessive CCS is added. The impedance variation indicates that a CCS content of 9% can relatively effectively improve the electrical resistivity of SAC mortar at different hydration ages, which reflects the improved continuity of the pore structure and reduced ion diffusivity.

#### 3.4.2. Electrical Resistivity

**Figure 13:**
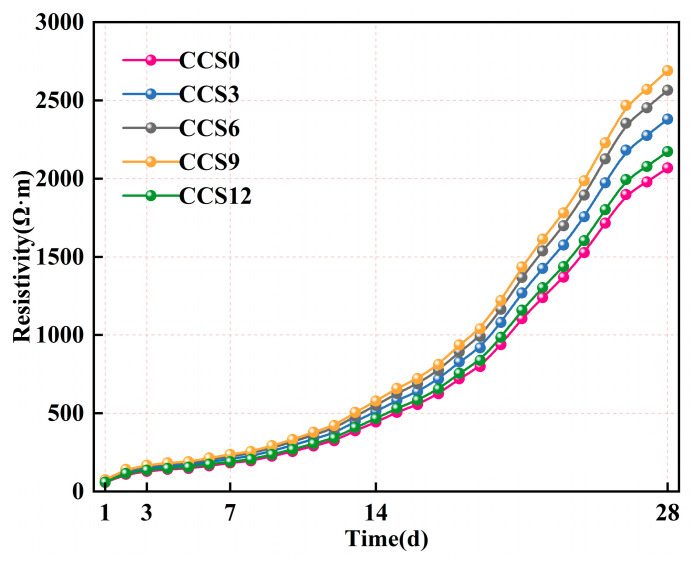
Resistivity of different mortar specimens.

Figure 13 shows the resistivity of the SAC system with different CCS doping levels as a function of curing age. The resistivity of the SAC system rises continuously with longer curing periods, suggesting that the pore structure of the CCS -doped SAC system is continuously refined with increasing curing age, and the internal ion transport pathways of the SAC system are gradually reduced. At the same curing period, the resistivity of the SAC system continuously reduces with CCS doping levels ranging from 0% to 9%, and begins to decrease when the CCS doping level reaches 12%. At any curing age, the resistivity of the SAC system with a 9% CCS doping level is greater than that with other CCS doping levels, indicating the existence of a relatively suitable CCS doping level that can ensure the densification of the pore structure and hinder internal ion transport. This may be because the high Ca(OH)_2_ content in CCS can accelerate the hydration reaction and promote the generation of dense hydration products such as C-(A)-S-H gel and AFt within the SAC system. These hydration products can effectively occupy the internal capillaries the SAC system, thereby reducing pore connectivity and hindering the conduction between ions [48]. The SAC system with 12% CCS doping exhibits a decrease in resistivity, possibly because the excessive CCS introduces an excessive amount of calcium-containing phase. These calcium-containing phases do not fully contribute to the hydration reaction within the SAC system, leading to uneven microstructure and increased local pore connectivity, which in turn hinders the improvement of resistivity.

At 1 day, the resistivity of all SAC systems was low, indicating that continuous pore water dominated the SAC systems at this age, and that the SAC systems were not fully hydrated. The resistivity of the SAC systems with CCS doping levels of 0–12% were 57.17 Ω·m, 65.74 Ω·m, 70.89 Ω·m, 74.32 Ω·m, and 60.03 Ω·m, respectively. Compared with the SAC systems without CCS, the resistivity of the SAC systems with CCS doping increased to varying degrees. This may be because the addition of CCS accelerated the initial hydration reaction process of the SAC systems, leading to the rapid generation of early hydration products and blocking some of the internal pore channels of the SAC system. At 3 days, the resistivity of all SAC systems continued to increase, and the differences between SAC systems with different CCS doping levels became more pronounced. The resistivity of the SAC systems with CCS doping levels of 0–12% were 128.91 Ω·m, 148.25 Ω·m, 159.85 Ω·m, 167.59 Ω·m, and 135.36 Ω·m. At this age, the SAC systems exhibited rapid formation of hydration products, and the initially interconnected capillaries transformed into a more tortuous pore network. At 28 days, the resistivity of all SAC systems reached its maximum, reflecting a highly refined pore structure and reduced ion mobility within the SAC systems. The resistivity of the SAC systems with CCS doping levels of 0–12% were 2069.15 Ω·m, 2379.53 Ω·m, 2565.75 Ω·m, 2689.90 Ω·m, and 2172.61 Ω·m. The SAC system with 9% CCS content maintained the highest resistivity throughout the 1–28 d hydration period, indicating that this CCS content provided a certain amount of calcium, thereby enhancing the early hydration reaction rate without causing excessive CCS particle aggregation.

During the 1–28-day curing period, the resistivity of the SAC system doped with CCS was consistently higher than that of the SAC system without CCS, highlighting its role in regulating the overall hydration behavior and microstructure development of the SAC system. At a CCS doping level of 9%, CCS provided sufficient active calcium to the SAC system and enhanced the internal hydration reaction, while maintaining the balance of the SAC solid–liquid system, which was beneficial for the uniform formation and maturation of hydration products. The SAC-CCS system exhibited a dense and continuous solid framework, low pore connectivity, and limited ion transport pathways [50].

### 3.5. Durability

#### 3.5.1. Chloride Ion Penetration Resistance

**Figure 14:**
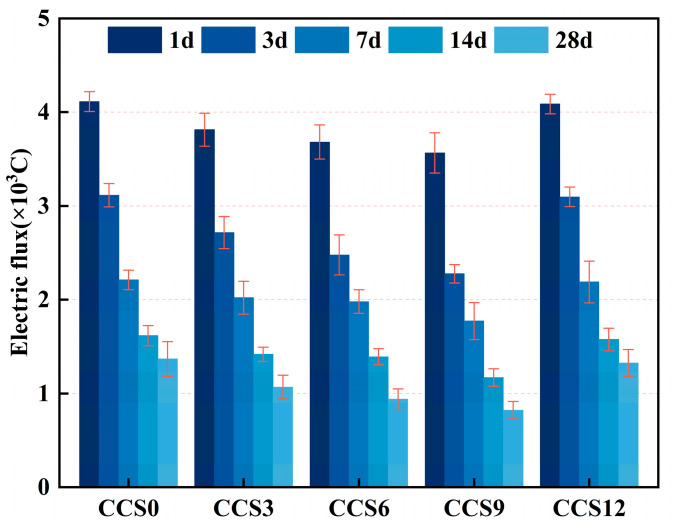
Electrical flux of different mortar specimens.

Figure 14 shows the measured electric flux of SAC mortar with various CCS contents at various curing ages. Two trends are observed from the Figure: first, for all mortar specimens, the electric flux decreased significantly with the prolongation of curing period (from 1 d to 28 d), indicating that the resistance to ion transport improved rapidly as hydration proceeded; second, at the same curing age, the electric flux reduced initially and then grow as CCS content increased, which suggests that the minimal electric flux was achieved at an appropriate CCS dosage. Among all the CCS dosage schemes, CCS9 exhibited the smallest electric flux, implying that the lowest electric charge passed through it and it performed best in restricting ion transport. Appropriate CCS dosage can provide additional Ca^2+^/OH^−^ and fine filler particles to exert the synergistic effects of chemical activation and micro-filling, refine the pore size distribution, reduce the connected porosity, thereby hindering ion migration and lowering the electric flux [48]. In contrast, excessive CCS in CCS12 would dilute the active clinker components and might leave a relatively high proportion of unreacted fine particles or poorly bonded zones; this would maintain or even increase the connected pathways for ion transport, thus increasing the measured electric flux.

At 1 d, the electric flux of all mortar specimens reached the maximum values, which were 4113 C for CCS0, 3813 C for CCS3, 3682 C for CCS6, 3565 C for CCS9, and 4087 C for CCS12, respectively. This reflects the open and highly connected pore network as well as the high ionic pore solution conductivity at the early stage. The dominant factors were pore connectivity and pore solution conductivity, rather than the newly formed small amount of gel phase; the differences among mortar specimens with different CCS contents were distinguishable but not significant. Notably, the electric flux of CCS9 showed a measurable reduction compared with CCS0, which indicates that even the early-stage micro-filling and the presence of additional Ca^2+^/SO_4_^2−^ from CS could accelerate the initial formation of precipitates, thereby partially hindering ion migration. However, CCS12 exhibited a higher electric flux than CCS0 at 1 d, which is consistent with the imbalance in particle packing, i.e., the reduced proportion of reactive binders led to insufficient early-stage pore blocking. The charge transport at the early stage was governed by pore solution conductivity and the open capillary pathways; moderate CCS addition could reduce the charge transport by providing nucleation sites and exerting the early-stage filler effect [51].

At 3 d, the electric flux of all mortar specimens decreased significantly compared with that at 1 d, with values of 3115 C for CCS0, 2716 C for CCS3, 2477 C for CCS6, 2277 C for CCS9, and 3097 C for CCS12, respectively. The ranking of electric flux for specimens with different CCS contents became clearer, with CCS9 exhibiting the minimum measurement and CCS0 the maximum. At this stage, primary phases such as AFt increased rapidly and began to block the capillary channels. Appropriate CCS content enhanced this densification through two pathways: first, micro-filling reduced the large capillary pores and increased the tortuosity; second, the interaction between CCS-derived Ca/alkalinity and active SiO_2_/Al_2_O_3_ accelerated the formation of secondary gel, thereby blocking the transport pathways [39]. These factors jointly reduced the connected porosity and lowered the ion migration rate, leading to a marked reduction in the electric flux of CCS9. In contrast, CCS12 still lagged behind, because the reduced content of active cementitious materials and the possible water competition effect limited the net production of pore-blocking gel at this stage. At 28 d, the electric flux of all mortar specimens reached the minimum quasi-stable values, which were 1368 C for CCS0, 1068 C for CCS3, 938 C for CCS6, 821 C for CCS9, and 1324 C for CCS12, respectively, indicating that the microstructure had matured, and long-term hydration and secondary reactions had significantly reduced the transport pathways. CCS9 exhibited the lowest electric flux, which was consistent with the optimized pore network and the relatively high electrical resistivity; the latter was ascribed to the abundant C-(A)-S-H and stable AFm/AFt aggregates that reduced the ion mobility. The electric flux of CCS0 was greater than that of CCS9 because it lacked the advantages of filler/secondary reaction; the performance of CCS12 was inferior to that of CCS9 because excessive replacement reduced the total reactivity of the binder and might maintain a relatively high proportion of connected pores or weak interfaces, thus facilitating ion transport over a longer period [52]. These trends are consistent with previously reported relationships among porosity, electrical resistivity and charge transport in blended cement systems.

**Figure 15:**
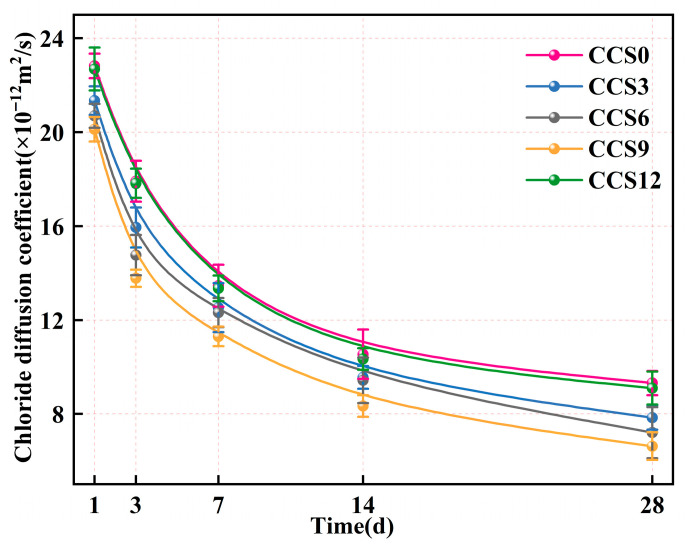
Chloride ion diffusion coefficient of different mortar specimens.

Figure 15 shows the chloride diffusion coefficient (CDC) of SAC mortar samples with various CCS contents at various curing ages. The CDC of all mortar specimens reduced monotonically with the growth of curing period. This trend reflects the enhancement of chloride ion penetration resistance of the mortar specimens as hydration proceeded. The key mechanism resides in the sustained hydration of the cementitious phase, which generates hydration products such as C-S-H gel to fill capillary pores and refine the pore structure. At any given age, the diffusion coefficient of CCS9 was lower than that of CCS0, indicating that incorporating a certain amount of CCS into SAC can suppress chloride ion diffusion. This may be attributable to the physical filling role of CCS, which densifies the SAC system and inhibits chloride ion migration [53].

At 1 day, the CDC of SAC system mortar samples with 0–12% CCS were all relatively high. This is because the SAC system is only partially hydrated at the initial stage, leading to a large number of capillaries and microcracks in the SAC system, which creates favorable channels for chloride ion diffusion. The diffusion coefficients of each group were as follows: 22.82 × 10^−12^ m^2^/s for CCS0, 21.34 × 10^−12^ m^2^/s for CCS3, 20.70 × 10^−12^ m^2^/s for CCS6, 20.12 × 10^−12^ m^2^/s for CCS9, and 22.69 × 10^−12^ m^2^/s for CCS12. The diffusion coefficient of the SAC system with 9% CCS content is greater than that without CCS, which indicates that the appropriate amount of CCS incorporated into the SAC system can promote early pore refinement. At 3 d, the CDC diffusion of all mortar specimens reduced significantly, with values of 17.91 × 10^−12^ m^2^/s for CCS0, 15.95 × 10^−12^ m^2^/s for CCS3, 14.77 × 10^−12^ m^2^/s for CCS6, 13.78 × 10^−12^ m^2^/s for CCS9, and 17.82 × 10^−12^ m^2^/s for CCS12, respectively. Increasing the CCS content accelerates the process of the SAC system. The resulting and other hydration phases occupy the pores within the SAC system and increase its overall density. The diffusion coefficient of CCS9 was lesser than that of CCS0, demonstrate that at a certain concentration of CCS dosage, the synergistic influence between cement hydration and CCS reaction was the most significant, thus forming a denser microstructure. At 28 d, the values of each scheme were as follows: 9.31 × 10^−12^ m^2^/s for CCS0, 7.84 × 10^−12^ m^2^/s for CCS3, 7.20 × 10^−12^ m^2^/s for CCS6, 6.62 × 10^−12^ m^2^/s for CCS9, and 9.10 × 10^−12^ m^2^/s for CCS12, correspondingly. The CDC of all mortar specimens reached the minimum values. This was because the hydration reaction was almost comprehensive, and the continuous reaction of CCS further refined the pore structure, minimizing the effective diffusion pathways for chloride ions. Among all groups, CCS9 had the lowest chloride ion diffusion coefficient, followed by CCS6, CCS3, CCS12 and CCS0. This indicates that CCS9 was the optimal CCS dosage, which could maximize the chloride ion penetration resistance.

#### 3.5.2. Carbonation Depth

**Figure 16:**
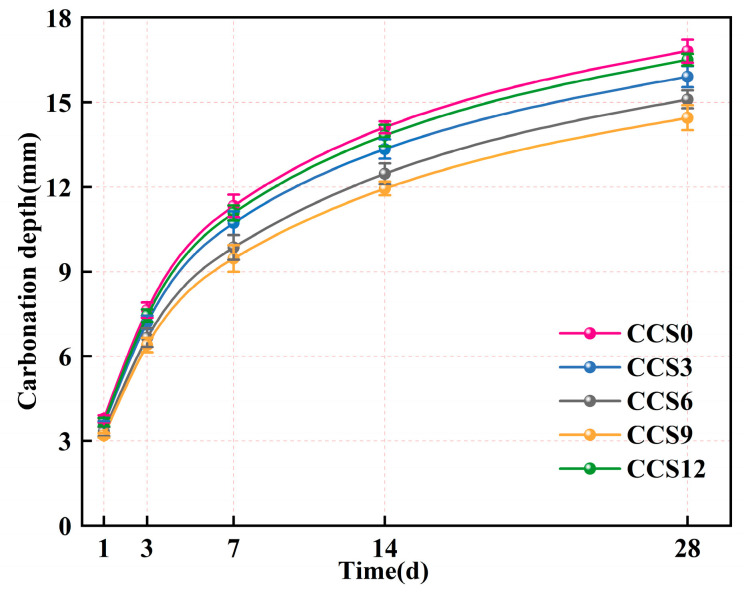
Carbonation depth in different mortar specimens.

As shown in Figure 16, the carbonization depth of the SAC system with CCS doping levels of 0–12% showed a significant increasing trend with increasing hydration age, while the overall growth rate was faster in the initial stages and slower in the final stages. From 1 day to 28 days, the carbonization depth of the SAC system at any CCS doping level continuously increased, indicating that the early-stage SAC system had high porosity and weak alkalinity, which facilitated rapid CO_2_ penetration. In the later stages of hydration, the SAC system generated a large amount of AFt and AFm phases, which gradually refined the pore structure and reduced CO_2_ diffusion rate. At any age, the carbonization depth of the SAC system without CCS was greater than that of the SAC system with CCS. The carbonization depth of the SAC system varied with different CCS doping levels, with the carbonization depth of the SAC system with 9% CCS being lower than that with 12% CCS. This may be because excessive CCS introduced additional fine pores and unreacted sulfate phases, thereby accelerating CO_2_ transport in the early hydration process of the SAC system. When an appropriate amount of CCS is incorporated into the SAC system, the SAC consumes free Ca(OH)_2_ through a hydration reaction, promotes the generation of secondary hydration products such as AFt/AFm, enhances the overall densification of the SAC matrix, stabilizes the alkalinity within the SAC system, and thus improves the overall resistance to carbonation [54].

At 1 day, the carbonation depths of the SAC systems with CCS doping levels of 0–12% were 3.79 mm, 3.60 mm, 3.33 mm, 3.20 mm, and 3.67 mm, respectively. At this age, the CCS doping level had a relatively small impact on the carbonation depth of the SAC system. The reaction of the SAC was in its initial stage, and its internal pore network remained highly open, allowing CO_2_ to quickly penetrate into the interior of the SAC system. Compared to the OPC system, the SAC system had relatively lower basicity and a limited Ca(OH)_2_ content, both of which reduced its resistance to carbonation. The SAC system with 9% CCS doping had a lower carbonation depth than those with other doping levels. This may be because CCS-derived CaSO_3_ reacted with the aluminates within the SAC system through hydration to form AFt. This early AFt crystallization made the SAC matrix more compact and reduced the connectivity between the capillaries within the SAC system [55].

As the age increased to 3 days, the carbonation depth of the SAC system continued to increase. The carbonation depths of the SAC systems with CCS doping levels of 0–12% were 7.64 mm, 7.20 mm, 6.66 mm, 6.40 mm, and 7.44 mm, respectively. When the CCS doping level was less than 9%, the incorporation of CCS improved the carbonation resistance of the SAC system. Appropriate CCS promoted the formation of secondary AFt and AFm within the SAC system, reduced porosity, and hindered the entry pathway of CO_2_. The carbonation depth of the SAC system without CCS was higher than that with CCS, possibly because the SAC matrix is generally porous and the Ca(OH)_2_ content within the SAC system is limited, failing to effectively neutralize the CO_2_ entering the SAC system. At a CCS doping level of 12%, the carbonation degree of the SAC system was relatively high, possibly due to the excessive sulfate phase increasing the capillary porosity within the SAC system, leading to smoother CO_2_ transport. Early-stage AFt generated within the SAC system may undergo carbonate-induced decomposition, generating carbonate-containing AFm, which further affects pore evolution and carbonation resistance [56].

The carbonization depth of the SAC system grew substantially from 3 d to 7 d, but the rate of rise decreased as the age increased from 7 d to 14 d and from 14 d to 28 d. For SAC systems with CCS doping increasing from 0% to 12%, the carbonization depth reached its maximum at 28 d, with depths of 16.81 mm, 15.90 mm, 15.10 mm, 14.45 mm, and 16.50 mm, respectively. The decreasing rate of increase in carbonization depth is likely attributable to the sustained hydration process between SAC and CCS, leading to the production of a denser AFt/AFm framework and C-(A)-S-H gel within the SAC system, and refining the pore structure of the SAC system [11]. The continuous hydration within the SAC system increases internal alkalinity by gradually releasing Ca^2+^ and OH^−^ ions, thereby enhancing its CO_2_ neutralization capacity. At 28 days, the carbonization depth of the SAC system with 9% CCS was less than that of the SAC system without CCS, indicating that an appropriate CCS content achieves a relative balance between pore refinement and alkalinity stability.

It should be noted that carbonation resistance alone does not fully represent long-term durability; however, when evaluated together with pore structure, electrical resistivity, and ion transport properties, it provides useful insight into the material’s resistance to aggressive environmental ingress.

### 3.6. SEM Analysis

**Figure 17:**
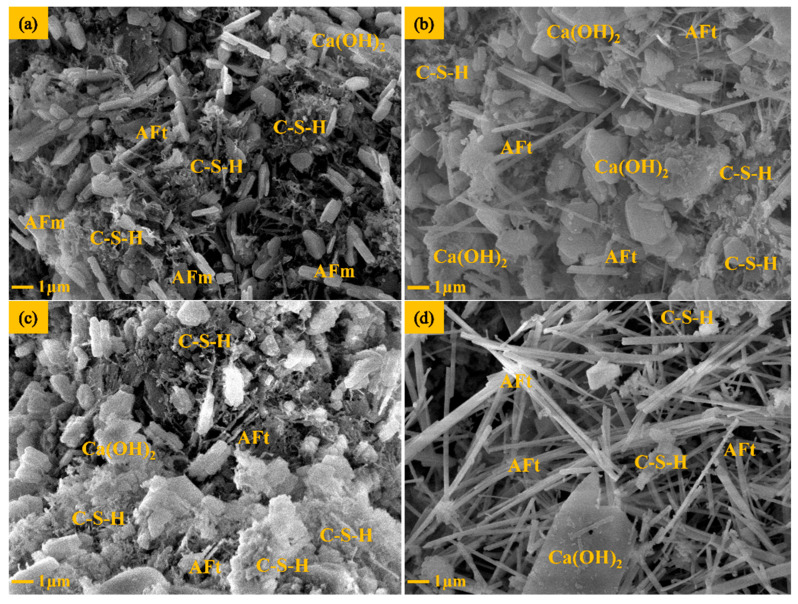

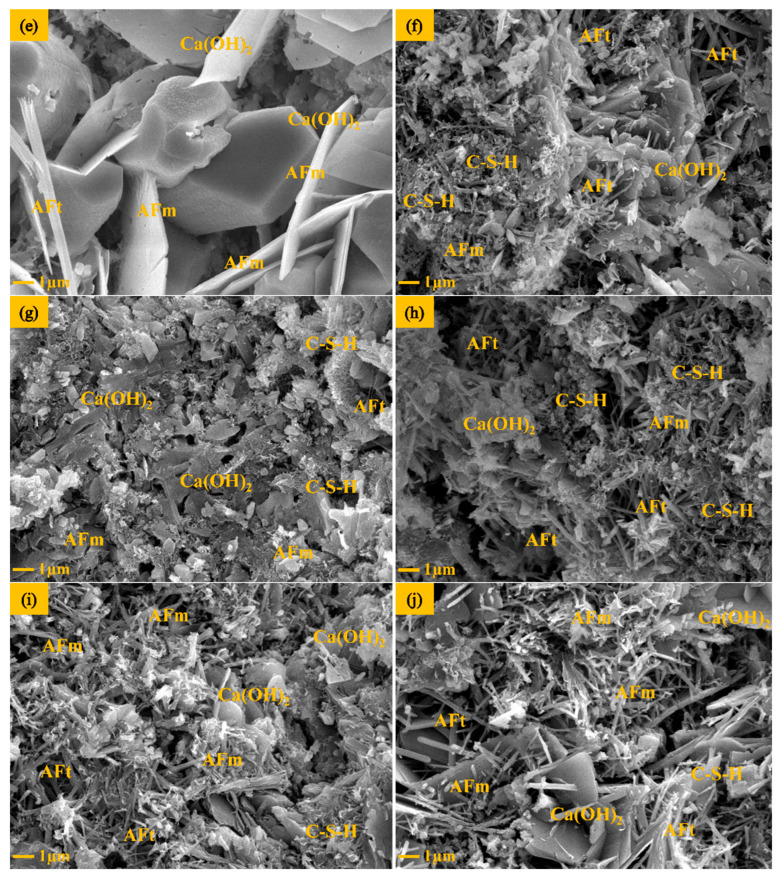
SEM images of SAC systems with different CCS doping levels. (**a**) CCS9-6 h; (**b**) CCS9-1 d; (**c**) CCS9-3 d; (**d**) CCS9-7 d; (**e**) CCS9-14 d; (**f**) CCS9-28 d; (**g**) CCS0-28 d; (**h**) CCS3-28 d; (**i**) CCS6-28 d; (**j**) CCS12-28 d.

Figure 17a–f show SEM images of the SAC system with a CCS content of 9% at 6 h, 1 d, 3 d, 7 d, 14 d, and 28 d, reflecting the changes in hydration products and microstructure over time after CCS incorporation into the SAC system. At 6 h, the microstructure of the SAC system mainly consists of a considerable amount of randomly oriented acicular AFt crystals, accompanied by a minor quantity of poorly crystallized gel. The matrix of the SAC system exhibits highly porous characteristics, indicating the typical reaction phenomena of rapid early hydration and AFt precipitation in the SAC system with appropriate CCS incorporation. At 1 d, the number and length of AFt crystals in the SAC system increase significantly, and plate-like hydration products begin to appear, partially filling the gaps between the AFt needle-like crystals and forming an interconnected network inside. From 3 d to 7 d, the microstructure of the SAC system becomes more compact. The AFt crystals in the SAC system are more uniformly distributed, and the dense AFt network coexists with a large amount of hydrated gel, increasingly embedding itself in the gel-like phase. The mutual growth of crystalline and amorphous hydration products in the SAC system leads to a finer internal pore structure, and most of the capillaries in the SAC system are effectively filled and exhibit high continuity, indicating that the hydration reaction of the SAC system is developing towards a stable and dense structure. From 14 d to 28 d, the microstructure of the SAC system is further densified. The AFt crystals in the SAC system are more tightly arranged, firmly bonded to the surrounding dense gel matrix, and form a uniform and stable microstructure. The secondary hydration and crystallization in the SAC system continue, leaving only a small number of isolated micropores in the structure of the SAC system. It is confirmed that the 9% CS doping content ensures that the SAC system achieves the best balance between AFt formation and long-term matrix densification during the hydration process.

Figure 17f–j show SEM micrographs of the SAC system with different CCS doping levels at 28 days. Incorporating 0–12% CCS into the SAC system reveals substantial differences in the morphology of phases of the microstructure. The SAC system without CCS exhibits a relatively loose matrix, primarily composed of numerous lamellar hydration products and some needle-like Aft crystals. Although the hydration reaction is largely complete, a small number of noticeable capillaries and microcracks remain, indicating that the pores within the SAC system are not completely filled. The SAC system with 3% CCS exhibits a denser microstructure than the SAC system without CCS. This may be because the additional Ca(OH)_2_ provided by CCS facilitates the continued hydration reaction and secondary precipitation of hydration products. The needle-like Aft crystals within the SAC system are embedded in a finer gel-like matrix, resulting in a more uniform and denser microstructure distribution. When CCS content is added to the SAC system at 6–9%, a large number of gel-like hydration products and a denser AFt crystal network are generated, effectively filling the pores within the SAC system. Compared with SAC systems without CCS and those with 3% CCS, fewer pores and microcracks are observed in the microstructure. This indicates that an appropriate amount of CCS can improve the basicity and calcium availability of the SAC system, promote the continuous hydration of C_4_A_3_Ŝ and belite phases in SAC, and facilitate the secondary reaction between dissolved Al_2_O_3_ and calcium compounds. Furthermore, the AFt crystals in the SAC system are more finely distributed, tightly interwoven with the surrounding gel matrix, resulting in a significantly refined pore structure and a continuous, uniform matrix, forming a dense SAC system framework. The strengthening of mechanical and durability characteristics of the SAC system with an appropriate amount of CCS is consistent with the microstructure observations. When the CCS content in the SAC system increases to 12%, signs of microstructure degradation begin to appear. Excessive needle-like AFt crystals in the SAC system exhibited localized aggregation, forming loose clusters. The SAC system also contained large plate-like crystals and unreacted or recrystallized phases. These hydration products all contributed to an increase in microporosity within the SAC system. This may be due to excessive CCS incorporation, leading to an oversupply of Ca^2+^ and OH^−^, disrupting the hydration equilibrium within the SAC system. This causes accelerated precipitation of hydration products in the initial stages, hindering the densification of the SAC matrix in later stages [57].

## 4. Conclusions

This study systematically explored the feasibility of incorporating CCS as an auxiliary material into SAC systems, focusing on its effects on workability, mechanical properties, hydration behavior, electrochemical characteristics, durability, and microstructure evolution. Based on the experimental results, the following conclusions were drawn:

(1) The incorporation of CCS significantly affects the workability of SAC. With increasing CCS dosage, both fluidity and setting time decrease. When the CCS dosage was controlled at 9 wt%, the SAC system exhibited higher compressive strength, lower drying shrinkage, and lower mass loss, indicating that an appropriate amount of CCS can effectively improve early hydration efficiency while maintaining volume stability.

(2) Electrochemical measurements of ER and EIS showed that the incorporation of CCS significantly altered the ion transport behavior within the SAC matrix. The SAC system containing 9 wt% CCS exhibited the highest ER and impedance values, indicating that its ion transport path was more tortuous and pore connectivity was reduced. An appropriate amount of CCS plays a crucial role in improving pore structure and inhibiting ion migration.

(3) Durability and microstructure reflect the effect of appropriate CCS addition. When the CCS content does not exceed 9 wt%, the chloride ion permeation resistance is significantly increased, manifested as a decrease in electrical flux and diffusion coefficient. SEM analysis shows that CCS promotes the uniform distribution of hydration products and enhances pore filling, thereby forming a denser and more continuous microstructure.

This study indicates that a 9 wt% dosage is considered the optimal dosage, and CCS can effectively serve as a functional supplementary material in the SAC system. The practical application of CCS-modified SAC systems still faces some challenges, and its long-term durability and sustainability assessment require further systematic research. Future research should focus on establishing a predictive model for the chloride ion permeation resistance life of CCS-SAC composites and assessing the environmental sustainability of CCS-SAC composites through quantitative analysis of three key indicators: material cost, energy consumption, and CO_2_ emissions.

## Figures and Tables

**Table 1:** Primary chemical constituents of SAC and CCS (wt%).

Materials (wt%)	CaO	SiO_2_	Fe_2_O_3_	Al_2_O_3_	MgO	SO_3_	TiO_2_	K_2_O	P_2_O_5_
SAC	39.54	13.47	2.65	22.25	0.212	17.43	1.01	0.409	0.243
CCS	90.82	3.10	0.80	1.80	0.09	3.288	0.065	0.002	0.035

**Table 2:** Main performance parameters of SAC and CCS.

Types	Density(g/cm^2^)	Specific Surface Area (m^2^/kg)	Compressive Strength (MPa)			Flexural Strength (MPa)		
			6 h	1 d	28 d	6 h	1 d	28 d
SAC	3.10	400	15	30	42.5	3.5	5.5	6.5
CCS	2.53	340	-	-	-	-	-	-

**Table 3:** Cement mortar sample mix proportion.

No.	Specimens	Cementitious Materials (%)		W/B	B/S
		SAC	CCS		
A1	CCS0	100	0	0.5	1:3
A2	CCS3	97	3		
A3	CCS6	94	6		
A4	CCS9	91	9		
A5	CCS12	88	12		

## Data Availability

The original contributions presented in this study are included in the article. Further inquiries can be directed to the corresponding author.
